# Gold Foil

**Published:** 1859-10

**Authors:** 


*Gold Foil.*—We received, a few weeks since, an eighth of an ounce of No. 4 gold foil, manufactured by Dr. D. S. Chase, of Augusta, Georgia. It is soft and tough, and easily compressed into very solid masses—properties all important to the dentist in filling teeth. In point of excellence, it is equal, we think, to any we have ever used.

